# Bushing Wear Prediction of High-Speed Press Conditions

**DOI:** 10.3390/ma19122614

**Published:** 2026-06-17

**Authors:** Alibek Yuldoshev, Inseo Kim, Joonhee Park, Junhee Chung, Taeyoung Im, Naksoo Kim

**Affiliations:** Department of Mechanical Engineering, Sogang University, Seoul 04107, Republic of Korea; alibek@sogang.ac.kr (A.Y.); adr0322@sogang.ac.kr (I.K.); yory11@sogang.ac.kr (J.P.); junhee2079@gmail.com (J.C.); ssk06328@naver.com (T.I.)

**Keywords:** bushing wear, archard wear model, finite element analysis, profilometry, high-speed press system

## Abstract

High-speed press systems operate under severe dynamic loading conditions, where bushing components are subject to accelerated wear that directly affects system reliability and maintenance cost. Despite extensive studies on bearing wear in automotive and aerospace applications, wear behavior under high-speed press conditions remains insufficiently explored. This study proposes a wear prediction model that integrates experimental measurements with finite element analysis (FEA). A key hypothesis is that bushing wear under high-speed press conditions can be accurately described by an extended Archard wear model incorporating contact pressure distribution and shaft misalignment effects. A controlled experimental setup was developed to replicate real operating conditions. Wear profiles were measured using high-resolution profilometry, while corresponding contact pressure distributions were obtained via 3D FEA simulations. Model parameters were calibrated using a subset of experimental data and validated against independent test cases. The proposed model demonstrates strong predictive capability, achieving an RMSE of 0.98 μm and an MAE of 0.57 μm across the 30-min calibration cases under the average (AVG) load-cell calibration. The extended formulation captures the asymmetric wear patterns induced by misalignment and resolves the high-pressure peak underestimation observed in the plain Archard baseline.

## 1. Introduction

Wear mechanisms are commonly classified into four types [1]: abrasive, adhesive, fatigue, and corrosive wear. Among these, adhesive wear is particularly relevant to sliding contacts. It originates from surface irregularities that create contact at discrete asperities [2]. At these points, localized temperature rises promote adhesion between mating surfaces. The combined action of stress and relative sliding then causes shear fracture within the bearing material. The detached material either transfers to the counter-surface or escapes as wear debris. As a result, rotational precision and overall system efficiency decline. This problem is especially critical in high-speed press machines.

High-speed mechanical presses are the principal machines for high-volume sheet-metal stamping, blanking, and forming. They convert the continuous rotation of a motor-driven flywheel into a reciprocating ram motion through a clutch, crankshaft, and connecting rod. The defining operating parameter is the stroke rate: conventional mechanical presses run at roughly 30–200 strokes per minute, high-volume production presses operate at about 120–300 SPM, and precision high-speed stamping machines reach 600–1400 SPM or higher. To sustain these rates the stroke length is kept short, typically a few tens of a millimeter. The press capacity ranges from tens to a few thousand tons and is delivered only near bottom dead center, so the drivetrain transmits a sharp load pulse on every stroke. These conditions impose a severe duty on the crankshaft bushings. Each stroke applies a cyclic radial load that is reacted through the crankshaft-bushing interface. The contact slides and oscillates under this load, the cycle repeats millions of times, and substantial frictional heat is generated. Consequently, at high stroke rates bushing overheating and wear, together with the associated loss of clearance, increasing vibration, and degraded stamping accuracy, become primary factors limiting machine life. Under such wear conditions, large-sized bushings endure extreme dynamic loads. Their eventual failure leads to substantial production downtime, since replacement is both costly and time-consuming.

Therefore, the life prediction of press machines is important issue of today’s engineering. Accordingly, scholars have developed several models to predict wear. Among them, the Archard wear model is the most widely used model [3,4]. It relates wear volume to normal load, sliding distance, and material hardness. In parallel, finite element methods (FEM) have proven reliable for capturing dynamic loads and contact pressures. Building on these foundations, Schmidt et al. [5] developed a 3D transient FEA framework for tilted shaft-bushing bearings in automotive turbochargers. Their model showed good convergence across mesh qualities and captured the evolution from line contact to parabolic wear patterns. Teng et al. [6] extended this approach by incorporating velocity-dependent friction coefficients through custom ABAQUS subroutines. Wang et al. [7] went a step further, coupling a mixed lubrication model with the Archard law for dynamically loaded journal bearings. They showed that nodal contact pressures from FE simulations accurately govern both the spatial pattern and the magnitude of early-stage wear. Despite these methodological advances, however, FEM-based wear simulations remain computationally constrained. Most are limited to short operating periods. Experimental validation, by contrast, requires tests lasting tens to hundreds of hours [8]. Therefore, Lin et al. [9] reported a similar issue: coupled lubrication-wear calculations involve repeated cyclic iterations, which quickly become prohibitively expensive for long-duration analysis. This temporal mismatch between simulation and experiment represents a fundamental challenge in wear prediction research as they presented. A parallel challenge exists on the experimental side as well. Conventional tribometers, such as pin-on-disk or ball-on-disk configurations, cannot reliably reproduce the line-contact behavior and clearance sensitivity of journal-bushing pairs [10]. Dedicated bushing-spindle test rigs are therefore required for representative characterization. However, even with such rigs, the existing literature still lacks sufficient data on the wear behavior of large bushings under the extreme cyclic loading unique to high-speed press machines.

To address this gap, the present study presents a physics-based wear prediction framework for copper alloy bushings under high-speed press conditions, which originally serves as the foundational stage of a broader research program aimed at developing comprehensive life estimation of press machine systems. Although previous scholars have validated the Archard model as a reliable framework for life cycle prediction in various applications that differ in principle from high-speed press systems, the present study draws upon their experimental methodologies, measurement protocols, and model development strategies as foundational background for the current work. Accordingly, this study is structured in two sequential stages. The first stage focuses on experimental characterization that conducts controlled accelerated wear tests at multiple durations and acquiring high-resolution wear profiles through profilometry to build a reliable empirical database of bushing wear behavior. The second stage develops an extended Archard-based wear model calibrated against the contact pressure distributions derived through FEA and validates its predictive capability against independent experimental data.

A critical methodological requirement in implementing this two-stage approach lies in correlating spatially resolved experimental wear measurements with the corresponding contact pressure distributions obtained through finite element contact analysis. Whereas Yin et al. [11] proved that wear model coefficients calibrated from 30-min accelerated bench tests could reliably predict full-scale engine durability, validating the use of short-duration experiments for long-term wear estimation. They employed an amplification factor approach to extrapolate single-cycle wear computations over multiple cycles, thereby reducing computational cost while preserving cumulative wear trends. The present study adopts a similar validation approach. It uses short-duration accelerated tests to establish predictive model coefficients but applies a different calibration strategy. Rather than simulating the operation cycle by cycle, static FEA-derived contact pressure distributions are directly correlated with experimentally measured wear profiles from accelerated tests. However, whereas Yin et al. validated their approach at a single operating duration against engine durability data, the present work extends this concept through accelerated wear tests conducted at three durations, namely, 30, 60, and 90 min at constant 300 rpm. It is hypothesized that wear model coefficients derived from short-duration accelerated tests at three-time cycles can more reliably predict wear at longer durations without recalibration.

As the main objective of the study, the life cycle of bushings under high-speed press operating conditions requires a wear model that correlates contact pressure distributions with progressive material removal over repeated loading cycles. The Archard wear model is the most widely adopted physics-based framework for this purpose, owing to its clear mechanistic foundation linking normal load, sliding distance, and material hardness to material removal [8,12]. Simultaneously, Archard formulas also work well with metals [12]. However, its original formulation estimates total volumetric loss under uniform contact assumptions, which cannot directly capture the spatially varying wear depth profiles observed in cylindrical bushing geometries where contact pressure is inherently non-uniform [3]. Kim et al. [13] addressed a similar limitation. They restructured the Archard equation from volumetric loss to directional wear depth, expressing it as $\delta =kP^{a}N^{b}$. The added load and cycle exponents capture nonlinear hardening effects. Calibrated against accelerated tests on simplified specimens, their model predicted full-scale product wear with a maximum deviation of 12.9% and $R^{2}$ above 0.94. Building on this work, the present study reformulates the Archard model as a spatial function: $h\left(x\right)=K^{*}p{\left(x\right)}^{\alpha }$. Here, the uniform normal load is replaced by the spatially resolved contact pressure p(x) obtained from FEA. The lumped coefficient $K^{*}=ks/H$ combines the wear coefficient, sliding distance, and material hardness. These quantities are treated as constants for a given test condition. The dimensionless exponent α characterizes the nonlinear relationship between wear depth and contact pressure. Specifically, α=1 recovers the classical Archard formulation, while α≠1 accounts for pressure-dependent wear mechanisms typical of misaligned journal-bushing interfaces. This spatial, nonlinear extension enables the model to capture the asymmetric wear morphologies that uniform-pressure models fail to reproduce. The need for nonlinear pressure dependence is further supported by Haneef et al. [14], who pointed that the wear coefficient varies significantly with the instantaneous contact state and cannot be reasonably treated as constant under severe contact conditions, particularly when surface asperities and contact area evolve dynamically during operation. The coefficients $K^{*}$ and α are subsequently determined through inverse calibration against experimentally measured wear profiles and their corresponding FEA pressure distributions. Inverse calibration of wear model parameters against indirect or sparse measurements has been demonstrated as an effective strategy by Haneef et al. [14], who calibrated Archard’s wear coefficient against measured signals to compensate for factors not explicitly resolved by the simulation.

By implementing this inverse calibration strategy, the proposed model effectively bridges the gap between empirical measurements and theoretical pressure distributions. It leads to a more comprehensive characterization of wear parameters under specific industrial conditions. This calibration approach further ensures that the model can capture the highly localized and nonlinear material removal rates that are often lost in conventional volumetric calculations. Accordingly, the primary objective of this research is to bridge the gap between short-term numerical simulations and long-term experimental validation which establishes a physics-informed framework for bushing wear in high-speed press systems. This study extends the Archard wear model in two ways. First, it adds a spatial term using contact pressure distributions from finite element analysis. Second, it introduces a nonlinear pressure dependence that accounts for shaft misalignment. The remainder of this paper is organized as follows. Section 2 describes the experimental methodology that covers the design of the accelerated wear test rig, the high-resolution profilometry measurements, and the 3D finite element simulation in ABAQUS used to extract nodal contact pressures. This section also details the mathematical formulation of the extended Archard model, and the inverse calibration strategy used to determine its coefficients. Finally, Section 3 and Section 4 present the experimental results and discussions, offering a comprehensive analysis of wear profile evolution across multiple durations and evaluating the model’s performance.

## 2. Methodology

### 2.1. Experimental Setup and Procedures

An experimental system was developed to analyze the wear behavior of bushings under realistic operating conditions. The system was designed to replicate the actual mechanical interaction between the crankshaft and bushings while enabling precise load control and continuous monitoring. The bushing specimens were fabricated from a special bronze casting alloy, the mechanical properties and chemical composition of which are shown in Table 1, with dimensions of 30 mm inner diameter, 40 mm outer diameter, and 50 mm length. To ensure experimental consistency, new bushings were used in each test. A crankshaft rotating at 300 rpm within the bushing pair was employed to reproduce the dynamic contact conditions representative of real press operation. The rig reproduces the press contact at a crankshaft speed of 300 rpm, equivalent to approximately 300 strokes per minute; the drive-train and specimen specifications are summarised in Table 2. Because direct long-duration testing on the production press is impractical, the present rig reproduces the governing tribological variables, namely the line-contact geometry, the radial load magnitude, and the sliding speed, at a fixed rotational speed of 300 rpm rather than the full reciprocating press cycle. This accelerated configuration yields measurable wear within minute-scale tests while preserving the contact conditions relevant to the press bushing.

The main components of the experiment are described in Table 2. The drive unit consisted of a Mitsubishi Electric AC servo motor controlled via an MR-J4 driver and configured through MR Configurator2 software (SW1DNC-MRC2-E, Ver. 1.21X). The 5 kW rated motor was driven by three-phase power and operated under a computer-based control system. Power transmission was achieved through a pulley-belt system, with a 63.5 mm pulley on the motor shaft and a 254 mm pulley on the crankshaft, forming a 4:1 reduction ratio. Accordingly, the motor operated at 1200 rpm while the crankshaft rotated at 300 rpm.

Two brackets were used to fix the bushings, designed to prevent rotation. Each bracket had two holes of 21 mm diameter on top for load application through load cells. A total of four load cells were installed, each applying 100 kgf for a total of 400 kgf. Metal discs were inserted between bolts and load cells to prevent damage during loading. TDK ferrite cores were attached to each load cell cable to minimize electromagnetic interference from the motor. Load signals were acquired through an Arduino-based data acquisition system and transmitted to a computer for real-time storage via a Python 3.11-based program. All experiments were performed at constant rotational speed for durations of 30, 60, and 90 minutes. After each experiment, bushing inner surface wear was measured using a Mitutoyo SJ-410 profilometer. Measurements were performed across the entire inner surface including top and bottom for precise spatial distribution analysis. The principal instruments and their suppliers were as follows: the AC servo motor (model HG-JR503) and MR-J4 driver (Mitsubishi Electric Corporation, Tokyo, Japan); the four compression-type load cells (model CSMN-1T, 1 t rated capacity, 100 kgf preload each, Curiosity Technology, Paju, Republic of Korea); the EMI-suppression ferrite cores (TDK Corporation, Tokyo, Japan); the data-acquisition microcontroller (Arduino Uno, Arduino, Italy); and the contact-stylus profilometer (model SJ-410, Mitutoyo Corporation, Kawasaki, Japan).

**Figure 1:**
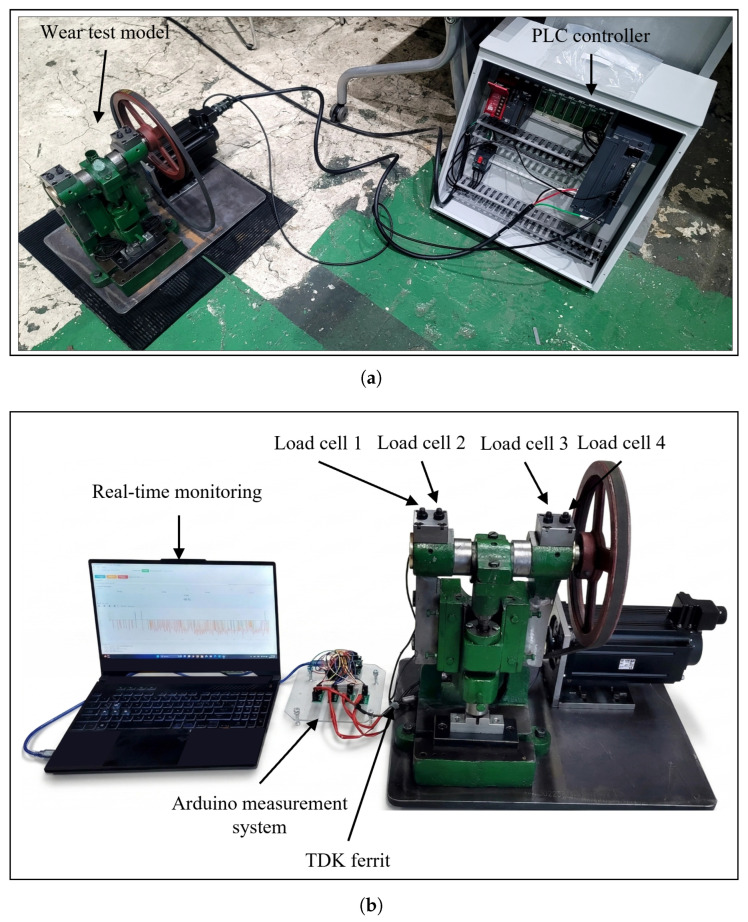
General configuration of the experimental and data acquisition systems: (**a**) Global view of the wear test model connected to the Programmable Logic Controller unit; (**b**) Detailed Data Acquisition setup featuring the Arduino measurement system, real-time monitoring interface, and EMI-shielded signal cables.

Prior to the tribological evaluations, a systematic assembly of the experimental rig was conducted to ensure structural integrity and kinematic accuracy. The global integration of the wear test model with the dedicated Programmable Logic Controller (PLC) unit for motor speed regulation and operational safety is illustrated in Figure 1a. The drive system’s operational parameters, including rotational direction and velocity profiles, were verified using the MR Configurator2 software environment.

**Figure 2:**
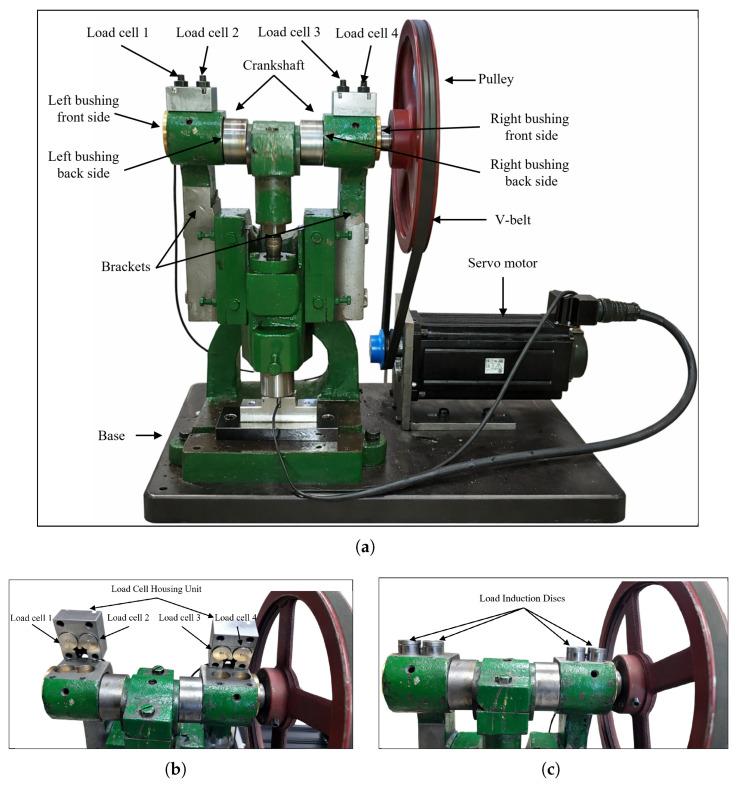
Detailed configuration of the accelerated bushing wear test rig: (**a**) Load cells (LC1–LC4), crankshaft, left and right bushings, supporting brackets, V-belt drive system, and servo motor mounted on a rigid base; (**b**) Load Cell Housing Units with integrated sensors in an open configuration; (**c**) Application of Load Induction Discs for uniform force transmission to the transducers.

The assembly process for the test specimens was executed according to the strict technical protocol to minimize experimental errors. Initially, the test bushings were integrated into the supporting brackets. To prevent rotational displacement or spinning of the bushing within the housing during high-torque conditions, an interference fit was established by precisely aligning the diameters of the bracket and the bushing. Subsequently, the bracket assemblies, containing the pre-installed bushings, were mounted onto the central crankshaft and secured using high-tensile bolts onto a rigid base. A 254 mm pulley connected via a V-belt to the servo motor was installed to facilitate the drive mechanism. This procedure was critical to ensure coaxial alignment and to minimize initial frictional resistance, as proper alignment was mandatory to prevent accelerated wear or catastrophic seizure of the crankshaft-bushing interface. To monitor the mechanical loads and tribological behavior in real-time, a high-precision data acquisition (DAQ) system was developed. The comprehensive configuration of the measurement system, including the Arduino-based DAQ unit and the real-time Python monitoring interface, is used as shown in Figure 1b. The normal and frictional forces were recorded using four high-precision load cells, which were securely mounted within a custom-designed Load Cell Housing Unit as given in Figure 2b. These specialized mounting fixtures, with dimensions of 45 × 50 × 25 mm, were installed above the bushings to accommodate the transducers. Each assembly incorporated two load cells, which were interfaced with custom-designed Load Induction Discs (Figure 2c). The size of the discs is at ⌀21 mm, thickness 4 mm, respectively. These discs serve as primary force transmitters, ensuring uniform load distribution and preventing localized stress concentrations or mechanical overloading of the sensors. Prior to testing, all load cells were subjected to a rigorous calibration procedure to ensure measurement fidelity. Furthermore, to mitigate electromagnetic interference (EMI) from the high-power electric motor, TDK ferrite cores were installed on the signal cables of each load cell as previously presented in Figure 1b.

These ferrite components function as passive electromagnetic filters, effectively improving the signal-to-noise ratio (SNR). All transducers were interfaced with an Arduino-based controller, enabling high-frequency data acquisition and efficient logging of experimental parameters. To maintain the reliability and repeatability of the experimental data, a rigorous calibration and loading sequence was followed. Before initiating the drive motor, a static load of 100 kgf was applied to each load cell and verified through the monitoring software. This initial loading phase was performed with the V-belt disconnected. This step was essential to isolate the system from parasitic radial loads induced by belt tension, which could otherwise skew the baseline measurements and compromise data integrity. Following the calibration phase, the V-belt was re-engaged, and the formal wear test was conducted at a constant rotational speed of 1200 rpm, with all tribological data being continuously logged for subsequent analysis. It should be noted that all wear experiments were conducted under dry contact conditions, without the use of lubrication oil. This approach was intentionally adopted to investigate the intrinsic wear behavior of the bushing material and to capture the worst-case tribological conditions [4]. Under actual high-speed press operation, the crankshaft-bushing interface is typically lubricated. The dry condition adopted here does not reproduce the nominal service state, but the dry condition is selected deliberately as an accelerated and worst-case bound. Removing the lubricant film suppresses the hydrodynamic and boundary-lubrication effects that would otherwise lower the wear rate. This maximizes the contact-pressure-driven material removal that the present model is designed to capture and compresses the test duration required to obtain measurable wear. The reported wear magnitudes therefore represent an upper bound rather than the in-service rate, and the quantitative effect of lubrication is identified as a limitation addressed in future work.

In this study, a total of 34 bushings were subjected to controlled wear testing, organized into 17 independent experimental runs. To ensure the reliability of the experimental results and to prevent cross-contamination caused by residual wear debris, two bushings representing the left and right sides were consistently used simultaneously during each test. After the completion of each test, these bushings were completely replaced with new specimens, thereby eliminating external influences and maintaining the accuracy of subsequent measurements. The same operating condition was repeated to assess test repeatability. The 30 min condition was reproduced across 8 independent runs including 3 failed tests, which constitute the calibration dataset used in Section 4 as a reliable data. The 60 min and 90 min conditions were each performed 6 and 3 times, respectively, to extend the duration range. The five-run repeatability established at 30 min tests, together with the consistent diagonal wear pattern observed at all durations, supports the reliability of these longer-duration measurements.

**Figure 3:**
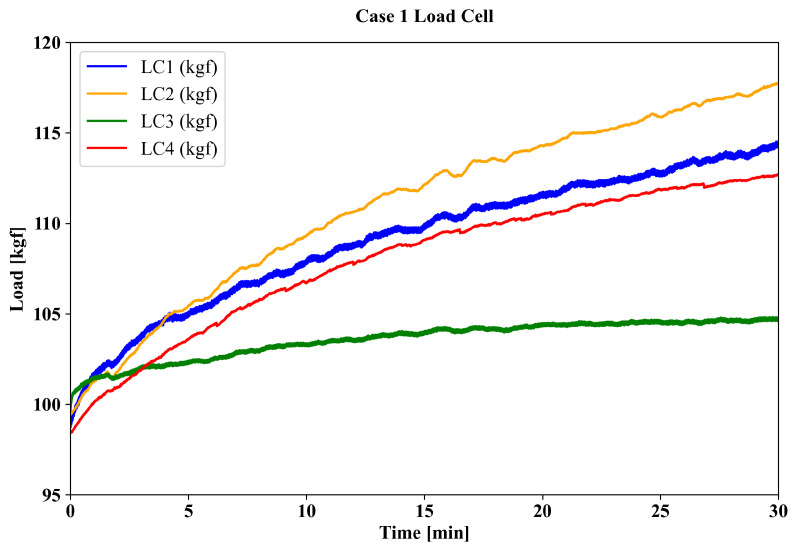
Representative load-cell recordings for Case 1 for the 30 min test.

Representative load-cell recordings acquired during Case 1 for the 30 min test are presented in Figure 3. The four sensors, denoted LC1, LC2, LC3, and LC4, were each pre-loaded to their nominal target of 100 kgf prior to belt engagement. Once crankshaft rotation was initiated, all four channels exhibited a gradual, monotonic increase throughout the test interval, reaching final values in the range of 105–118 kgf at 30 min. The progressive load rise is attributed to thermal expansion at the contact interface and to the progressive evolution of the crankshaft-bushing contact geometry under dry friction conditions. To convert the time-resolved signals into a single representative loading state for the finite element simulation, the time-averaged value of each load cell over the full test duration was adopted and used as the input boundary condition for the FE model described in Section 2.3. The resulting AVG contact-pressure field was subsequently paired with the profilometry-measured wear depths to calibrate the spatial Archard model defined in Section 2.4, as detailed in Section 4.

### 2.2. Wear Profile Measurement

**Figure 4:**
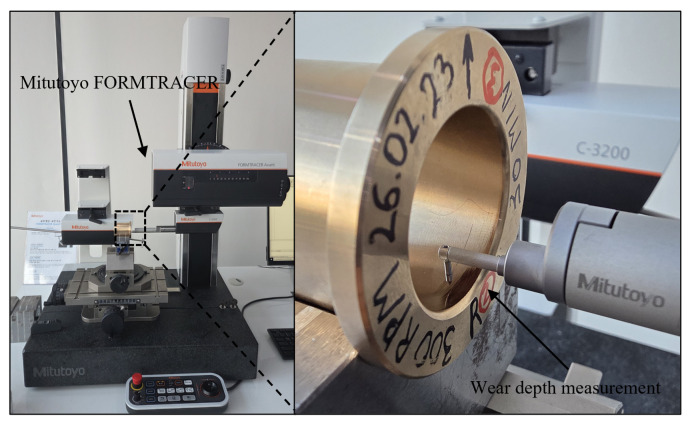
Wear profile measurement using the Mitutoyo SJ-410 contact-stylus profilometer.

Macroscopic inspection of the tested specimens revealed a non-uniform wear distribution. The localized wear at axial edges is consistent with the stress redistribution mechanisms as explained by Teng et al. [6]. The edge-concentrated loading phenomena, as indicated by Colbert et al. [15], demonstrated that even slight angular misalignment between shaft and bushing axes produces severe edge loading that dramatically reduces component life. As wear progresses, initial high-stress contact zones expand, reducing peak contact pressure while redistributing load over a larger area. This stress evolution is further influenced by shaft misalignment, which concentrates contact at bushing edges. This behavior was consistently observed across multiple samples and indicates that the loading and contact conditions were not uniformly distributed along the axial direction. Due to the inherent difficulty in directly measuring wear on the internal cylindrical surface of the bushings, a high-precision Mitutoyo SJ-410 contact stylus profilometer was employed (Figure 4). This technique enabled accurate acquisition of surface profile data along the axial direction and allowed detailed characterization of localized wear features [16]. As a result, the wear distribution and surface topography were captured with high resolution, particularly in critical regions where wear is most evident.

### 2.3. FE Simulation

**Figure 5:**
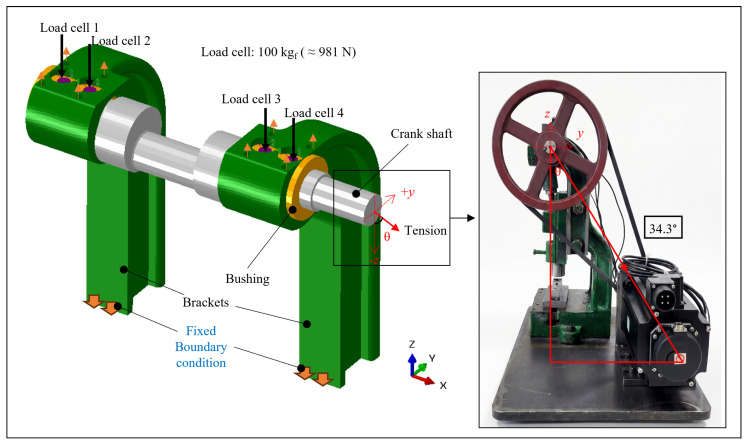
FE simulation model and the actual experimental setup at the nominal belt inclination angle of 34.3° measured from the vertical. The model illustrates the boundary conditions, load-cell positioning, and the applied tension vector.

A three-dimensional finite element model of the test rig was developed in ABAQUS/Standard 2020 to extract the contact pressure distribution required by the wear model in Section 2.4. The model included the two brackets, the two bushings, the crankshaft, and four rigid load-cell bodies, as illustrated in Figure 5. A consistent MPa-mm-t-s unit system was adopted throughout the analysis. The *X*-axis was aligned with the crankshaft axial direction, and the *Z*-axis was aligned with the vertical loading direction.

The bushings and the contact region of the crankshaft were discretized with linear hexahedral C3D8R elements to ensure accurate resolution of the contact pressure. The brackets and the remainder of the crankshaft were meshed with quadratic tetrahedral C3D10 elements to accommodate their geometric complexity. The complete model contained approximately 93,000 nodes and 59,000 elements. All components were treated as homogeneous, isotropic, and linearly elastic materials. The bushings were assigned the cast bronze properties characterized in Table 1, with a Young’s modulus of 100 GPa and a Poisson’s ratio of 0.35. The steel components were assigned the properties of S45C steel, with a Young’s modulus of 190 GPa and a Poisson’s ratio of 0.27.

To evaluate the mesh independence of the finite element contact solution, the simulation was repeated using three mesh sizes: 5 mm, 7 mm, and 9 mm. The maximum contact pressure (CPRESS) was selected as the mesh-sensitivity indicator because the FEA-derived contact-pressure field is the primary input for the subsequent wear model. Following the approach used in previous numerical tribological studies [5,13], mesh dependence was assessed by comparing the key output quantity obtained from different discretization levels. The 5 mm mesh produced a stable and physically reasonable contact-pressure distribution, with the pressure concentration located at the dominant wear region observed experimentally on the right bushing. Therefore, the 5 mm mesh was selected for the subsequent finite element and wear analyses.

**Figure 6:**
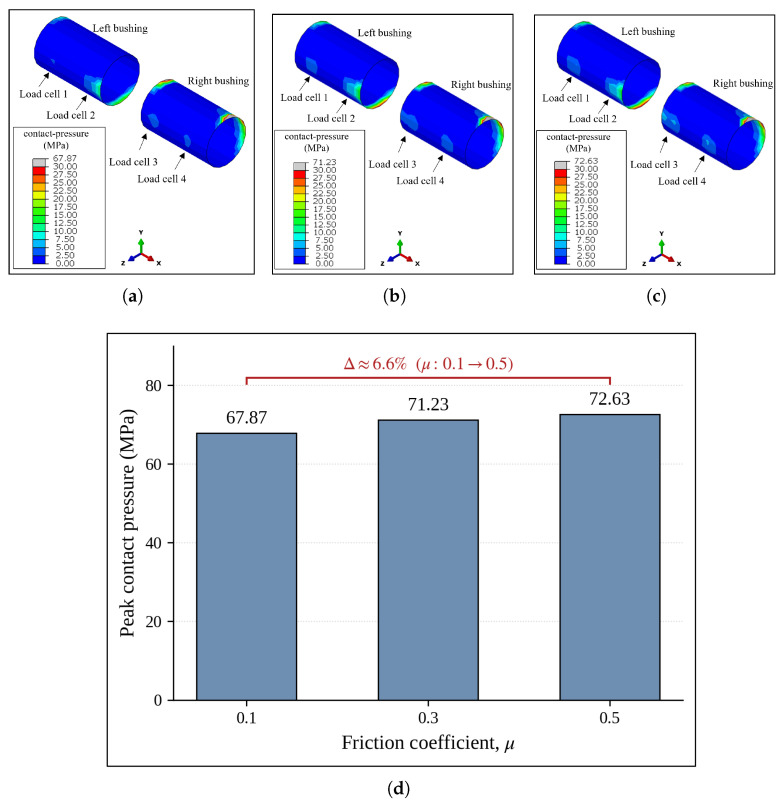
Effect of the friction coefficient on the predicted contact pressure for Case 1: contact-pressure contours at (**a**) μ=0.1, (**b**) μ=0.3, and (**c**) μ=0.5, shown on a common 0–30 MPa scale, and (**d**) the corresponding peak contact pressure. The location and pattern of the contact-pressure concentration are identical for all three values, and the peak varies by only 6.6%.

Surface-to-surface contact was specified at the bushing-crankshaft and bushing-load-cell interfaces. A Coulomb friction coefficient of 0.5 and a hard normal pressure-overclosure relation were applied at these interfaces. The bushing-bracket interfaces were modeled as tied contact to reproduce the experimental interference fit. A geometrically nonlinear static analysis was then performed under three superimposed loading actions. First, prescribed vertical displacements were applied at each load-cell reference point and calibrated such that the FE reaction force matched the experimentally measured 100 kgf, equivalent to approximately 981 N, at each sensor. Second, a vertical bracket load of 1962 N was applied to represent the static weight transmitted from the upper press tooling. Third, a belt tension of 1000 N was applied at the crankshaft reference point at an angle of 34.3° from the vertical, matching the measured belt geometry. The bracket mounting faces were fully constrained using the ENCASTRE boundary condition. The Coulomb friction coefficient of 0.5 is consistent with the worst-case dry condition adopted throughout this study [13,17,18]. Because the friction coefficient governs tangential traction at the interface, its influence on the contact-pressure field was quantified by repeating the Case 1 analysis at μ=0.1, 0.3, and 0.5 in Figure 6. The location and pattern of the contact-pressure concentration were identical for all three values, and the peak contact pressure varied by only 6.6% over this five-fold change in friction. The predicted contact-pressure distribution, and therefore the wear distribution, is thus essentially insensitive to the assumed friction coefficient.

The diametral clearance between the bushing and the crankshaft varied from 0.01 to 0.04 mm under both symmetric and asymmetric configurations. This variation was performed to investigate the effect of assembly tolerances on the contact pressure distribution. Following solver convergence, contact pressure was extracted along the bushing inner surface at 5 mm axial intervals.

### 2.4. Wear Model Formulation

Wear models are commonly classified into mechanical and phenomenological categories. In this study, the Archard wear model was adopted because of its practical applicability and broad use in journal-bearing wear prediction [19,20,21]. Although the original Holm–Archard formulation was developed for adhesive wear associated with asperity-level contact and plastic deformation, the model has since been widely extended to other wear conditions, including abrasive wear, fretting wear, and mixed wear mechanisms [22,23]. Therefore, the present study uses the Archard framework as the basis for relating the local contact condition between the crankshaft and bushing to the resulting wear depth.

The standard Archard wear model is written as follows [19]:

(1) $$V=\frac{KFs}{H},\;$$

where *V* is the wear volume, *K* is the dimensionless wear coefficient, *F* is the normal load, *s* is the sliding distance, and *H* is the hardness of the softer material.

In the present study, the experimentally measured quantity is the local wear depth along the axial direction of the bushing rather than the total wear volume. Therefore, Equation (1) is reformulated in terms of wear depth. By dividing the wear volume by the apparent contact area and expressing the normal load as contact pressure, the Archard equation can be rewritten as

(2) h=kps,

where *h* is the wear depth, *p* is the normal contact pressure, and *k* is a dimensional coefficient defined as K/H. Since the material hardness of the bushing is assumed to remain constant during the accelerated wear tests, *H* is incorporated into the coefficient *k*. This local formulation is consistent with previous finite-element-based wear studies, in which Archard’s law was applied point-by-point or node-by-node using local contact pressure values [5,7,9].

The sliding distance can be expressed as s=2πrN where *r* is the crankshaft radius and *N* is the number of rotation cycles. For a given test duration and rotational speed, the sliding distance is constant. Therefore, *s* can be incorporated into a lumped coefficient. In addition, the measured wear profiles show strong spatial variation along the axial direction of the bushing. Accordingly, the wear depth is expressed as a function of the axial coordinate *x*. To account for possible nonlinear pressure dependence under misaligned contact conditions, the spatial Archard model is formulated as

(3) $$h\left(x\right)=K^{*}\;p{\left(x\right)}^{\alpha },\;$$

where h(x) is the wear depth at axial position *x*, p(x) is the corresponding contact pressure obtained from the finite element analysis, $K^{*}=ks=Ks/H$ is a lumped dimensional coefficient, and α is a dimensionless pressure exponent. When α=1, Equation (3) reduces to the classical linear Archard pressure dependence. When α≠1, the model accounts for nonlinear pressure–wear behavior, which may occur under localized edge loading and shaft-misalignment-induced contact concentration.

The wear model in Equation (3) describes the relationship between contact pressure and wear depth. However, the model is limited in its ability to fully capture the actual wear behavior of bushings because wear progression is also influenced by local deformation and strain concentration occurring at the contact interface. In practical bushing systems, localized stretching and nonlinear deformation behavior may significantly affect wear evolution, which cannot be sufficiently represented by contact pressure alone. Therefore, a stretch ratio term was additionally introduced to incorporate the influence of local deformation into the wear model. Accordingly, the improved wear model is expressed as follows:

(4) $$h\left(x\right)=a_{0}+a_{1}\;p{\left(x\right)}^{k_{1}}+a_{2}\;p{\left(x\right)}^{k_{2}}\;\lambda {\left(x\right)}^{n},$$

where p(x) denotes the local contact pressure extracted from the FE analysis, and λ(x)=exp(ε(x)) represents the stretch ratio, where ε(x) is the corresponding maximum-principal logarithmic (true) strain extracted from the same FE solution. The coefficients $a_{0}$, $a_{1}$, $a_{2}$, $k_{1}$, $k_{2}$, and *n* are empirical model constants determined through parameter calibration. The coefficients $a_{1}$ and $a_{2}$ are dimensional scaling factors associated with the pressure- and deformation-dependent wear contributions, respectively, while $k_{1}$ and $k_{2}$ govern the nonlinear sensitivity of wear evolution to contact pressure. The constant term $a_{0}$ is introduced to compensate for geometric and wear-initiation variations among different bushings. In addition, the exponent *n* controls the nonlinear influence of the stretch ratio on wear progression.

In this study, wear behavior was predicted using both the original wear model in Equation (3) and the improved wear model in Equation (4), followed by a comparative evaluation of their predictive performance. The model coefficients were determined using a least-squares fitting approach to minimize the discrepancy between the experimental measurements and the model predictions. The predictive capability of each model was subsequently evaluated by comparing the predicted wear profiles with the experimentally measured wear profiles of the bushings. For quantitative model assessment, the coefficient of determination ($R^{2}$) was employed together with the root mean square error (RMSE) and mean absolute error (MAE). The corresponding evaluation metrics are defined as follows:

(5) $$\mathrm{RMSE}=\sqrt{\frac{1}{n}\sum_{i=1}^{n}{\left(h_{\exp,i}-h_{\mathrm{pred},i}\right)}^{2}},$$

(6) $$\mathrm{MAE}=\frac{1}{n}\sum_{i=1}^{n}\left|h_{\exp,i}-h_{\mathrm{pred},i}\right|,$$

where *n* is the number of axial data points used in the comparison, and the subscript *i* denotes each measurement point. RMSE reflects the overall prediction error with greater sensitivity to large local deviations, whereas MAE represents the average absolute difference between the measured and predicted wear depths. Therefore, RMSE is effective for evaluating localized prediction inaccuracies, while MAE provides an overall measure of the average prediction performance across the entire wear profile.

## 3. Results and Analysis

### 3.1. Experimental Wear Profile Analysis

**Figure 7:**
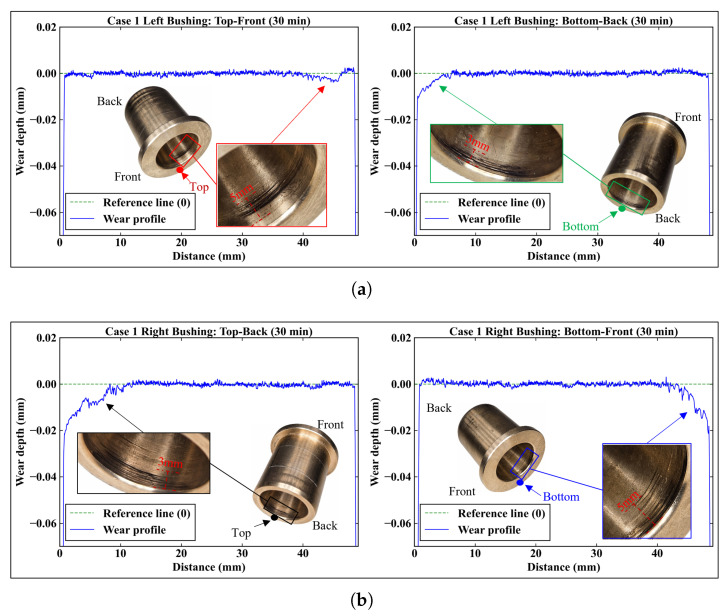
Worn profiles of the left and right bushings after 30 min of testing (Case 1): (**a**) left bushing—top-front and bottom-back traces; (**b**) right bushing—top-back and bottom-front traces. Optical insets show the worn bands on the inner surface.

**Figure 8:**
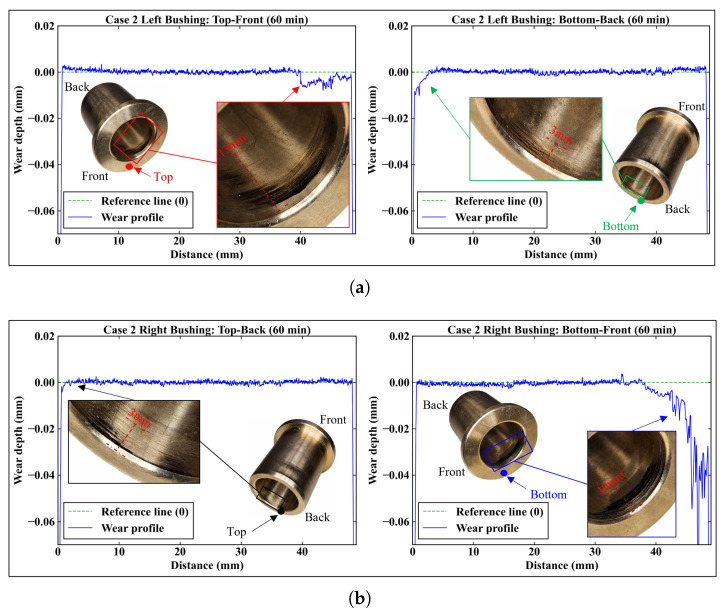
Worn profiles of the left and right bushings after 60 min of testing (Case 2): (**a**) left bushing—top-front and bottom-back traces; (**b**) right bushing—top-back and bottom-front traces. Optical insets show the worn bands on the inner surface.

**Figure 9:**
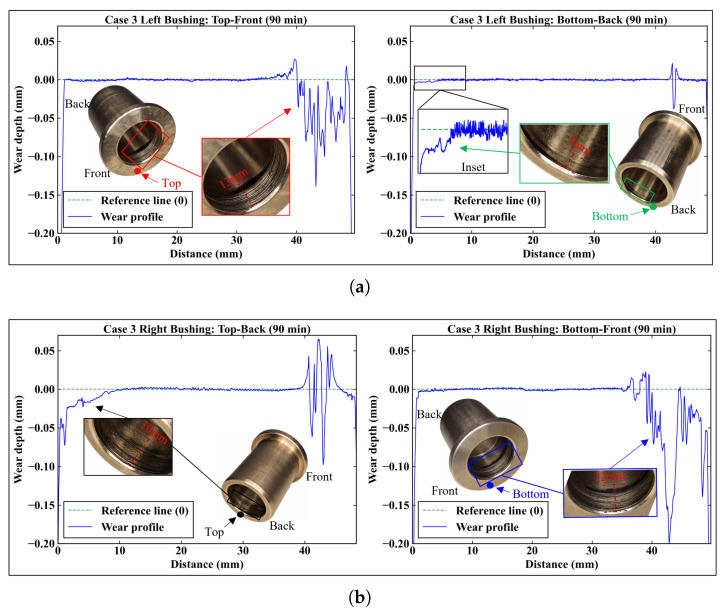
Worn profiles of the left and right bushings after 90 min of testing (Case 3): (**a**) left bushing—top-front and bottom-back traces; (**b**) right bushing—top-back and bottom-front traces. Vertical scale extended to −0.20 to +0.05 mm. Optical insets show the worn bands on the inner surface.

The axial wear profiles measured on the inner surface of each bushing are presented in Figure 7, Figure 8 and Figure 9 for test durations of 30, 60, and 90 min, hereafter referred to as Case 1, Case 2, and Case 3, respectively. These are raw measured profiles, and one representative profile was selected for each duration as the clearest example. Together they show how the worn region is distributed around the circumference of the bore and how it grows as sliding time increases from 30 to 90 min. A single profile per duration is sufficient for this, because the spatial pattern is the quantity of interest, not the test-to-test scatter. The averaged and segmented profiles are reported in Section 4, where they are also calibrated against the FEA results.

To capture the full circumferential and axial extent of the contact, two diametrically opposed measurement traces were acquired on every specimen: the top-front and bottom-back traces for the left bushing, and the top-back and bottom-front traces for the right bushing. This diagonal pairing reflects the contact geometry expected under a tilted-shaft condition, in which the upper and lower contact patches form on opposite axial extremities of the bushing rather than directly above one another.

Across all three cases, three features were systematically observed: (i) the central axial region of every profile remained within the noise band of the reference baseline, (ii) measurable material removal was confined to narrow zones adjacent to one of the two axial faces, at the front or at the back, and (iii) for every test duration, the deepest and widest worn band consistently appeared at the bottom-front trace of the right bushing. These observations indicate that the wear process at 300 rpm under dry contact was governed by a deterministic, geometry-driven contact distribution rather than by a uniform sliding mechanism. This behavior is the key prerequisite for the spatial reformulation of the Archard model proposed in Section 2.4.

#### 3.1.1. Case 1

After 30 min of operation, the inner surface of each bushing was profiled with an SJ-410 contact stylus profilometer of 0.001 μm resolution. Two diametrically opposed axial traces were measured per specimen to capture any contact asymmetry expected under a tilted shaft. The left bushing was sampled at the top-front and bottom-back, and the right bushing at the top-back and bottom-front. Across all four traces shown in Figure 7, the wear depth stays within ±0.005 mm of the baseline along about 90% of the measured axial length. This confirms that the running-in stage has only just begun to localize the contact. Figure 7a presents the left bushing. Its top-front trace is essentially featureless along the full 0–38 mm range. Only minor stylus noise appears, with a small upward deviation near the front face beyond 40 mm. The optical inset, however, reveals a faintly polished band about 5 mm wide near the front edge. Nascent contact has therefore formed even though the macroscopic depth stays below the plot resolution. The diagonally paired bottom-back trace shows a comparably mild response. A shallow deviation of about 0.003 mm appears at the entrance region between 0 and 5 mm, with a corresponding worn band of about 3 mm in the inset. The contact loads on the left bushing are therefore mild at this stage, with only nascent wear bands forming at the diagonally paired axial extremities.

Figure 7b shows the right bushing. Its top-back trace remains flat along the reference line. A narrow-worn band of about 3 mm appears at the back face, comparable in extent to the bands on the left bushing. The bottom-front trace, however, departs sharply from this regime. It develops the most evident wear of the entire dataset. A clearly defined local depression of about 0.025 mm forms between 37 and 46 mm, with an optically distinguishable worn band of about 5 mm. The right bushing therefore exhibits the same diagonal pairing as the left, here between the top-back and bottom-front traces. Combined with the markedly deeper wear of the bottom-front zone, this confirms that the 30 min wear distribution is not stochastic. It follows a deterministic, geometry-driven contact pattern, consistent with a slightly tilted shaft-bushing engagement. The vertical bracket loads transmitted through the load cells combine with the inclined belt tension at 34.3° from the vertical. Their resultant biases the contact patches toward diagonally opposite axial extremities, rather than forming a uniform line contact. This geometry concentrates wear at the bottom-front of the right bushing and provides direct experimental evidence of an asymmetric, edge-concentrated contact condition. Whether this initial pattern persists, broadens, or relocates as testing proceeds is examined in Cases 2 and 3.

#### 3.1.2. Case 2

Figure 8 shows the wear profiles of the left and right bushings obtained after 60 min of testing. The same four traces measured in Case 1 were re-acquired. Figure 8 shows that the diagonal wear topology established at 30 min remains intact. The four active wear zones still appear at the same diagonally paired locations, but the localized depressions have intensified in depth and broadened in axial extent. The central axial region of every trace continues to lie within the noise band of the reference baseline. Measurable material removal therefore stays confined to narrow zones near one axial face of each bushing. Figure 8a presents the left bushing. The top-front trace develops a shallow depression of small amplitude near 38–45 mm. Its optical inset reveals a worn band of about 13 mm, the widest single band recorded at this duration. The depth of the depression remains modest, but this band is 2.6 times wider than the 5 mm band seen at 30 min. The front-edge contact patch has therefore begun to spread inward along the bushing axis. The diagonally paired bottom-back trace, in contrast, remains close to the reference line. Only a minor deviation of about 0.005 mm appears at the entrance region between 0 and 5 mm. The corresponding worn band of about 3 mm in the inset is essentially unchanged from Case 1. The left bushing therefore wears asymmetrically along its own diagonal, with the top-front zone broadening laterally while the bottom-back zone stays nascent.

Figure 8b shows the right bushing. The top-back trace again exhibits only a shallow disturbance along its full length. A narrow worn band of about 3 mm at the back face matches its Case 1 morphology. The bottom-front trace, however, develops the most pronounced wear feature of the 60 min dataset. A deep, smoothly varying depression of about 0.060 mm forms between 38 and 46 mm, accompanied by a worn band of about 10 mm in the optical inset. The maximum wear depth has therefore more than doubled relative to Case 1. The axial width of the worn band has approximately doubled as well. The bottom-front of the right bushing persists as the dominant wear site, deepening and broadening in place rather than relocating. Read together with Figure 8a, the 60 min state shows that the asymmetric contact distribution identified at 30 min continues to govern the wear evolution. The early-stage contact patches act as preferential pathways for continued material removal rather than being redistributed by progressive mating of the two surfaces. The intensification is itself spatially asymmetric. The front-edge zones broaden from 5 mm to 13 mm on the left top-front and from 5 mm to 10 mm on the right bottom-front. The back-edge zones, however, remain at about 3 mm. Whether continued operation produces further axial broadening or transitions to depth-dominated wear progression at the same locations is examined in Case 3.

#### 3.1.3. Case 3

Figure 9 shows the wear profiles of the left and right bushings obtained after 90 min of testing. To accommodate the substantially larger amplitudes at this duration, the vertical scale was extended from ±0.020 mm to a wider window of approximately −0.20 to +0.05 mm. Two qualitatively new features distinguish Case 3 from the shorter durations. The maximum wear depth has increased by roughly an order of magnitude relative to Case 1. High-amplitude oscillatory deviations also emerge near the heavily worn axial extremities, including occasional positive spikes above the reference line. The diagonal wear topology established in Cases 1 and 2 nevertheless remains spatially intact, and the worn regions stay concentrated at the same axial extremities of each bushing. Figure 9a presents the left bushing. The top-front trace remains close to the reference level over the central 0–35 mm region. Near 38–46 mm, however, it shows abrupt fluctuations and sharp drops reaching approximately −0.10 to −0.15 mm. A worn band of about 13 mm is clearly resolved in the optical inset. The axial width of this band is essentially unchanged from Case 2, but its depth has deepened by roughly two orders of magnitude relative to the 60 min state. The front-edge contact patch on the upper surface of the left bushing has therefore transitioned from a broadening-dominated to a deepening-dominated wear regime. The diagonally paired bottom-back trace remains close to the reference line throughout the central region. The inset zoom highlights persistent low-amplitude deviations near the entrance at 0–5 mm that were already present in Cases 1 and 2. A positive spike is also observed at the front face, attributable to wear-debris adhesion on the worn surface. The upper extremity of the left bushing therefore acts as the dominant wear site on this side, while the bottom-back zone remains nascent.

Figure 9b shows the right bushing. The top-back trace remains essentially featureless along most of its length. Isolated positive and negative spikes appear near the front face, and a worn band of about 10 mm is visible in the optical inset. The bottom-front trace, however, exhibits the most apparent wear of the entire dataset. The wear depth approaches −0.15 to −0.20 mm, and the trace is dominated by oscillatory deviations exceeding 0.10 mm in localized bursts. The corresponding worn band extends over about 12 mm. Across all three test durations, this trace consistently emerges as the deepest and widest worn region. Its maximum wear depth increases from approximately 0.025 mm in Case 1, to 0.060 mm in Case 2, and to 0.15–0.20 mm in Case 3. The spatial location of this trace, at 37–46 mm, stays essentially unchanged throughout. The bottom-front of the right bushing therefore identifies the global maximum of the tribological response under the nominal 34.3° configuration.

Wear processes in sliding contacts are commonly described in terms of an initial break-in phase, in which mechanical deformation and asperity-level hardening dominate, followed by a steady-state phase in which the contact area stabilizes, and material removal proceeds at a more uniform rate [24]. Taken together, the three cases of the present study accordingly reveal an analogous two-stage wear progression at the dominant contact zones. Between 30 and 60 min, the worn bands grow primarily in axial extent at fixed axial locations. On the left top-front, the band broadens from 5 mm to 13 mm. On the right bottom-front, it broadens from 5 mm to 10 mm. The peak depths over this interval remain modest, with the right bottom-front rising only from 0.025 mm to 0.060 mm. Between 60 and 90 min, the wear progression switches regime. The axial widths stabilize, increasing only marginally to 13 mm on the left top-front and 12 mm on the right bottom-front. The peak depths instead increase sharply. The left top-front deepens from a few μm to 0.10–0.15 mm, and the right bottom-front deepens from 0.060 mm to 0.15–0.20 mm. The first interval is therefore a broadening phase, in which the worn bands grow in axial extent at fixed axial locations and the contact area progressively evolves. The second interval is a deepening phase, in which the same bands deepen by roughly an order of magnitude with little additional lateral spread, consistent with material removal proceeding at an established contact geometry. The persistence of the wear topology throughout the test, combined with the monotonic deepening at fixed axial locations, supports the central modeling assumption introduced in Section 2.4. The local wear depth can be expressed as a stable nonlinear function of a position-dependent contact pressure. A single set of model coefficients calibrated against the FEA-derived contact pressure distributions should therefore be capable of reproducing the wear evolution across all three cases.

### 3.2. FE Simulation Results

**Figure 10:**
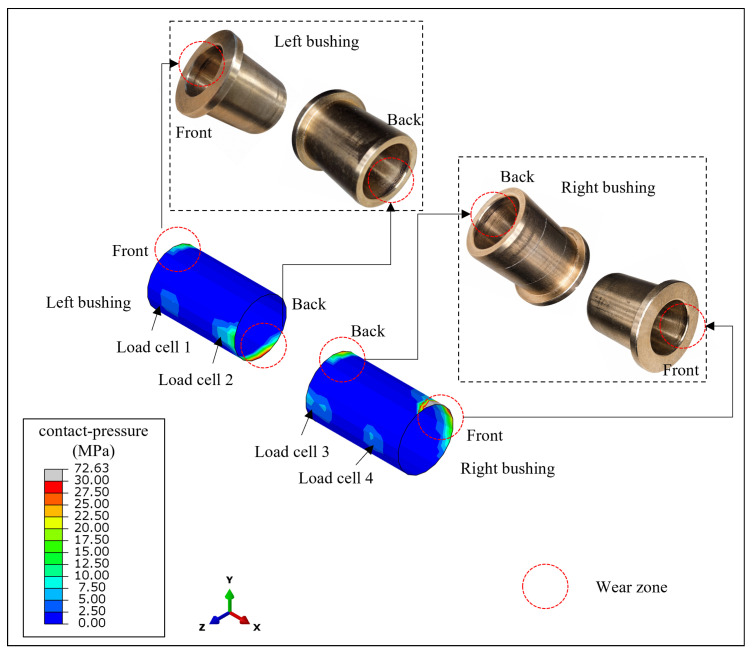
ABAQUS contact-pressure distribution and corresponding experimental wear marks on the left and right bushings for Case 1. Dashed red circles indicate the high-pressure zones and matching wear locations.

The wear morphology characterized in Section 3.1 is governed by the underlying contact pressure distribution between the crankshaft and the bushing inner surface. Figure 10 presents the FEA-derived contact pressure distribution on the inner surface of the left and right bushings for Case 1. The two bushings are mounted in mirror-image orientation within their respective brackets. The loaded axial extremity of the left bushing and the right bushing are positioned on opposite sides of the assembly. The color scale is clipped at 30 MPa to enhance the visibility of the contact bands. The global pressure maximum, 72.63 MPa, occurs on the right bushing, indicated by the saturated grey region.

Two qualitative features dominate the pressure field. First, contact is confined almost entirely to narrow bands at the axial extremities of each bushing, while the central region of the inner surface remains effectively pressure-free. Second, the loaded extremity of each bushing is diagonally asymmetric. A dominant contact patch develops at the lower portion of one axial face, that is, the back face on the left bushing and the front face on the right bushing. A smaller satellite patch develops at the upper portion of the opposite axial face. The four load cells deliver the radial preload through the rigid bracket adapters described in Section 2.3. The resulting contact patches are localized immediately on the inner surface adjacent to the loaded faces. This diagonal pairing on the FE pressure field is the direct mechanical counterpart of the diagonal worn band pattern characterized experimentally in Section 3.1, and the predicted wear zones marked with dashed red circles in Figure 10 coincide with the trace positions where the deepest wear was measured.

For both bushings, the pressure field exhibits a strongly axially asymmetric distribution. The central region of the bushing (x≈15–30 mm) carries pressures below 2 MPa, while pressure peaks one to two orders of magnitude higher emerge at the two axial faces. The left bushing develops peaks of 31.2 MPa at x=0 mm (back edge) and 19.1 MPa at x=50 mm (front edge), whereas the right bushing develops a moderate peak of 28.1 MPa at the back edge and a substantially higher peak of 72.6 MPa at the front edge. The peak-to-middle pressure ratio reaches approximately 20:1 on the left bushing and exceeds 45:1 on the right bushing, confirming that the contact patches occupy only narrow circumferential bands at the axial extremities, in agreement with the diagonally paired worn bands presented experimentally in Section 3.1.

The dominant front-edge peak of 72.6 MPa on the right bushing coincides spatially with the bottom-front trace identified in Section 3.1 as the deepest and most consistently worn region across all three cases. This co-location of the dominant FEA pressure peak with the dominant experimental wear site provides direct mechanical justification for the asymmetry observed in the wear measurements: the same loading geometry that produces edge-concentrated contact in the FE simulation also produces the diagonally paired wear pattern measured by profilometry.

## 4. Discussion

The experimental wear measurements presented in Section 3.1 were combined with the FE-derived contact pressure field discussed in Section 3.2 to calibrate the proposed spatial wear model. The calibration was performed under the average load-cell calibration condition, hereafter referred to as the AVG calibration. For the 30 min calibration cases, the time-averaged value of each load cell was used as the representative loading condition in the FE simulation, as described in Section 2.1. The corresponding bracket displacement was determined through the inverse procedure described in Section 2.3, so that the FE reaction forces matched the experimentally measured average load level. The resulting AVG contact-pressure field was then paired with the segment-wise wear depths obtained from profilometry. The coefficients $K^{*}$ and α were identified by least-squares fitting of Equation (3) using the combined left- and right-bushing dataset. The same AVG calibration dataset was also used to fit the extended pressure–strain wear model in Equation (4). This allowed the plain spatial Archard model and the extended formulation to be compared under the same loading condition. The dataset consisted of five 30-min cases, including one top trace and one bottom trace from each bushing. In total, 20 traces and 220 axial sample points were used for calibration. The calibrated coefficients and fitting metrics obtained under the AVG calibration are summarized in Table 3.

**Figure 11:**
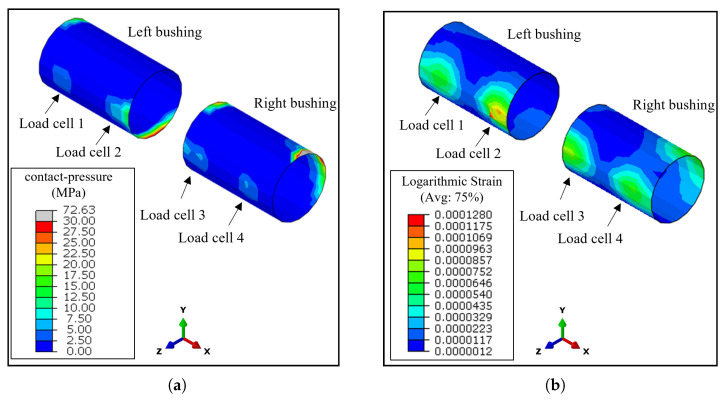
Spatial co-location of contact pressure and local strain for Case 1: (**a**) contact-pressure distribution and (**b**) maximum-principal logarithmic strain on the bushing inner surface. Both fields concentrate in the same loaded zones at the front edges, supporting the strain-coupling term in Equation (4).

The plain spatial Archard model converged to $K^{*}=0.609$ and α=0.929, with an overall RMSE of 1.42 μm and an MAE of 0.81 μm. Since α is close to unity, the average pressure–wear relationship follows the classical Archard tendency in the moderate-pressure region. However, this baseline model underestimated the peak wear at the highly loaded edge regions, especially at the bottom-front trace of the right bushing. To improve the prediction of this localized peak wear, the extended pressure–strain wear model in Equation (4) was applied: $h\left(x\right)=a_{0}+a_{1}p{\left(x\right)}^{k_{1}}+a_{2}p{\left(x\right)}^{k_{2}}\lambda {\left(x\right)}^{n}$. In this formulation, $a_{0}$ is the baseline wear offset that absorbs the running-in stage and the profilometer detection floor. The pair $\left(a_{1},k_{1}\right)$ defines the polynomial Archard-type pressure contribution, and the pair $\left(a_{2},k_{2}\right)$ sets the pressure scaling of the strain-coupling term. The exponent *n* controls how strongly the local stretch ratio λ(x)=exp(ε(x)) amplifies this contribution, with λ→1 in the small-strain limit. The extended pressure–strain model converged to $a_{0}=0.738\;\mu$m, $a_{1}=-1.116$, $k_{1}=2.095$, $a_{2}=1.210$, $k_{2}=2.075$, and n=0.9997. This reduced the RMSE from 1.42 μm to 0.98 μm and the MAE from 0.81 μm to 0.57 μm. The coefficient of determination increased from $R^{2}=0.558$ for the plain spatial Archard model to $R^{2}=0.787$ for the extended pressure–strain model. These results show that introducing the multiplicative stretch-ratio coupling improves the fitting accuracy, particularly in the heavily loaded edge-contact zones where the plain spatial Archard model underestimates the peak wear. The physical rationale for the stretch-ratio term is that, at the heavily loaded bushing edges, the contact is not purely elastic but involves localized plastic stretching of the near-surface material. This local deformation enlarges the real area of asperity contact and promotes the material detachment that underlies the Archard mechanism, so wear scales more steeply than contact pressure alone would predict. The maximum-principal logarithmic strain extracted from the same FE solution was adopted as the simplest scalar measure of this localized deformation. Other mechanical quantities, such as the local shear stress, the frictional (dissipated) work, or the strain energy density, could in principle play an equivalent role, and the present formulation does not claim that strain is uniquely superior to these alternatives. To substantiate this, the maximum-principal logarithmic strain field from the Case-1 analysis was examined alongside the contact-pressure field (Figure 11).

The two fields concentrate in the same loaded zones at the bushing front edges, confirming that high contact pressure and large local deformation are spatially co-located. The material at these edges therefore experiences both the highest pressure and the largest local straining, which together intensify the near-surface deformation and material removal beyond the level predicted by contact pressure alone.

**Figure 12:**
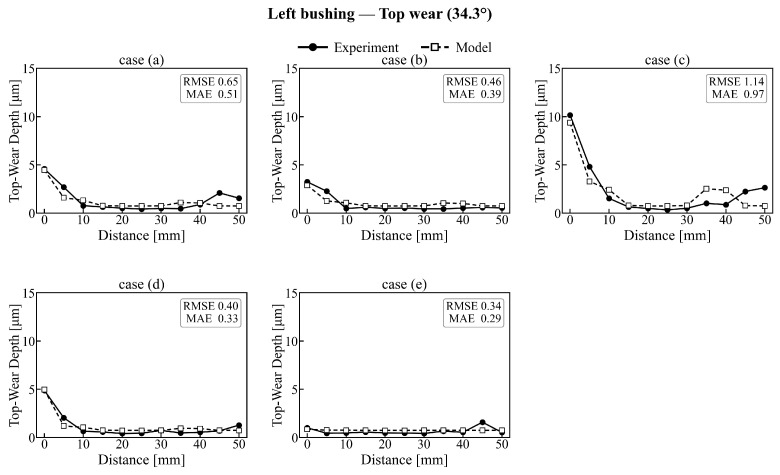
Top-trace wear of the left bushing under the AVG load-cell calibration at 34.3°. Panels (**a**–**e**) show the five 30 min cases. Filled circles denote experimental data, and open squares denote the prediction from Equation (4).

**Figure 13:**
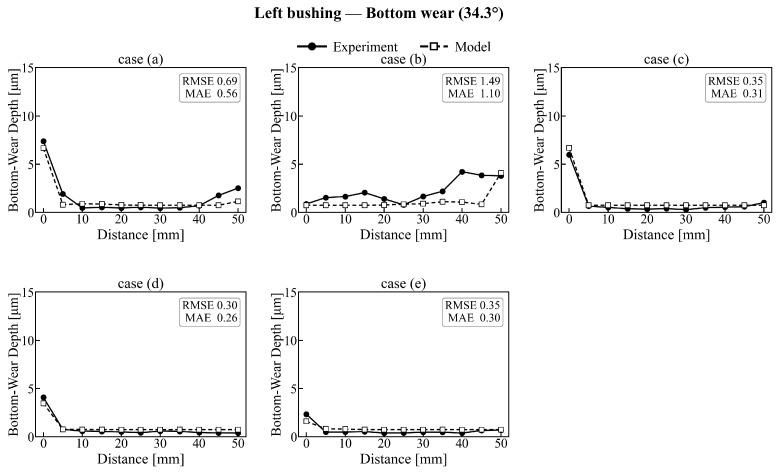
Bottom-trace wear of the left bushing for the same five cases shown in Figure 12. Panels (**a**–**e**) correspond to the five 30 min cases. The comparison is made under the AVG load-cell calibration at 34.3°. Filled circles and open squares represent experiment and Equation (4) prediction, respectively.

**Figure 14:**
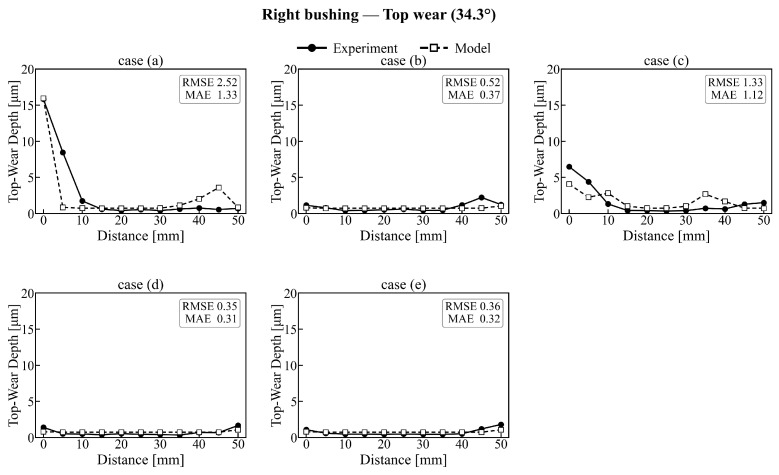
Top-trace wear of the right bushing under the AVG load-cell calibration at 34.3°. Panels (**a**–**e**) correspond to the five 30 min cases. Experimental and predicted values are shown by filled circles and open squares, respectively.

**Figure 15:**
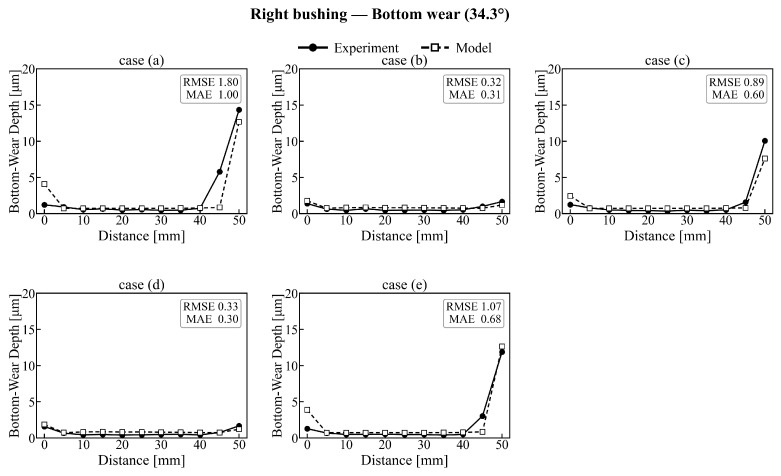
Bottom-trace wear of the right bushing, corresponding to the dominant front-edge wear region predicted by the FE contact-pressure analysis. Panels (**a**–**e**) show the same five 30 min cases as in Figure 12. Filled circles indicate experimental data, and open squares indicate Equation (4) prediction.

A direct comparison between the measured and predicted wear depths is presented in Figure 12, Figure 13, Figure 14 and Figure 15. The figures are arranged according to bushing side and trace position: Figure 12 shows the top trace of the left bushing, Figure 13 shows the bottom trace of the left bushing, Figure 14 shows the top trace of the right bushing, and Figure 15 shows the bottom trace of the right bushing. Each figure includes five independent 30-min calibration cases under the nominal belt angle of 34.3°. In all panels, filled circles denote the experimental wear depths measured by profilometry, while open squares represent the prediction obtained from the extended pressure–strain wear model in Equation (4).

Figure 12 presents the top-trace wear of the left bushing. This trace corresponds to a relatively mild-wear condition compared with the dominant edge-contact region of the right bushing. Therefore, it provides a useful test of whether the proposed model remains valid not only under severe edge loading but also under low-amplitude wear conditions. Across the five cases, the panel-wise RMSE ranges from 0.34 to 1.14 μm, indicating generally close agreement between the measured and predicted profiles. In most cases, the measured wear is concentrated near the back edge at x=0 mm, while the central region remains close to the baseline. The model reproduces this decreasing edge-to-centre trend well. Case (c) shows the largest wear amplitude in this trace, reaching approximately 10.16 μm at x=0 mm, compared with the predicted value of 9.37 μm. Cases (d) and (e) show the closest agreement, with RMSE values of 0.40 μm and 0.34 μm, respectively. Small unresolved deviations remain in cases (a)–(c), particularly near the opposite edge, where the simulated pressure is low. These deviations are likely associated with local running-in effects, surface irregularity, or small contact-state variations that are not fully represented by the static FE pressure field.

Figure 13 shows the bottom-trace wear of the left bushing. The model again follows the dominant experimental trend, with panel-wise RMSE values ranging from 0.30 to 1.49 μm. Similar to the top trace, the main wear in most panels appears near the back edge, whereas the central region remains nearly flat. Cases (c) and (d) show particularly good agreement, with RMSE values of 0.35 μm and 0.30 μm, respectively. Case (b) shows the largest deviation because the measured wear peaks on the front side, near x=40 mm (about 4.2 μm), while the model predicts only 1.07 μm at the same point. The model is built on a single, as-assembled static pressure field, so it cannot reproduce wear that comes from conditions specific to one specimen. Small differences between specimens, such as initial seating, local clearance, or running-in, can concentrate contact on the front edge and remove extra material that the static pressure field does not predict. In addition, once the front edge starts to wear, the contact patch shifts and the local pressure rises above the as-assembled value used in the model, so a static field tends to underestimate this late-stage edge wear. These effects are limited to this one heavily loaded specimen and do not change the good edge-peak agreement seen in the other four cases.

Figure 14 presents the top-trace wear of the right bushing. Compared with the left-bushing traces, this position includes stronger edge-localized wear in some cases. The panel-wise RMSE ranges from 0.35 to 2.52 μm. The model accurately captures the general edge-to-centre decay of the wear profile and maintains good agreement in the low-wear central region. Cases (d) and (e) show the closest agreement, with RMSE values of 0.35 μm and 0.36 μm, respectively, while case (b) is also well reproduced with an RMSE of 0.52 μm. The largest RMSE appears in case (a). However, the main edge peak itself is predicted accurately: the measured wear reaches approximately 15.86 μm at x=0 mm, while the model predicts 15.94 μm. Thus, the relatively high RMSE in this panel mainly originates from scatter in the surrounding low-wear points rather than from failure to capture the dominant peak. In case (c), the model reproduces the overall decreasing trend but underestimates the local edge peak, suggesting that some local contact amplification remains unresolved.

Figure 15 shows the bottom-trace wear of the right bushing, which is the most critical trace in the dataset. This trace corresponds to the dominant front-edge contact region identified in the FE contact-pressure analysis. In all five cases, the main wear peak appears near the front edge at x=45–50 mm, consistent with the location of the highest simulated pressure. The model follows the experimental trend well, with panel-wise RMSE values ranging from 0.32 to 1.80 μm. Cases (b) and (d) have relatively small wear amplitudes and are reproduced with low RMSE values of 0.32 μm and 0.33 μm, respectively. Cases (a), (c), and (e) show stronger front-edge wear, and the model captures the sharp increase near the loaded edge. In case (e), the peak agreement is especially close, with 11.89 μm measured and 12.64 μm predicted. In cases (a) and (c), the model slightly underestimates the front-edge peak, but the location and shape of the dominant wear zone are still reproduced. This result confirms that the strain-coupled term in Equation (4) improves the prediction of localized peak wear in the high-pressure edge-contact region.

Taken together, Figure 12, Figure 13, Figure 14 and Figure 15 demonstrate that the extended pressure–strain wear model improves the fitting accuracy while preserving the physical basis of the spatial Archard formulation. The plain spatial Archard model captures the general pressure–wear relationship, but it tends to underestimate localized peak wear at heavily loaded edges. By incorporating the multiplicative stretch-ratio coupling, Equation (4) better reproduces the diagonal wear pattern, the left–right bushing asymmetry, and the dominant front-edge wear of the right bushing. Across the 20 traces, the per-trace RMSE remains below 1.5 μm in most cases, and the overall fitting accuracy reaches $R^{2}=0.787$, RMSE = 0.98 μm, and MAE = 0.57 μm.

**Figure 16:**
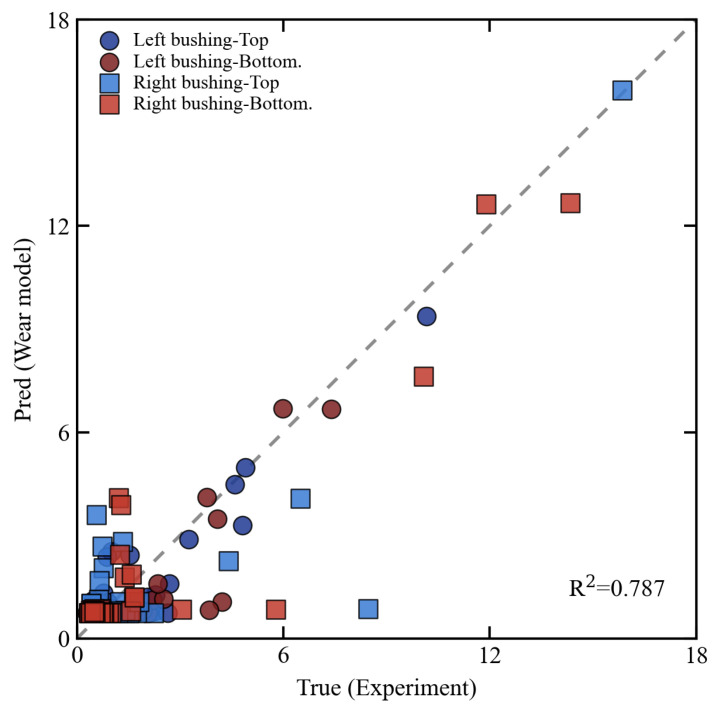
Parity plot of the predicted versus measured wear depth across all 220 sample points from the five 30-min calibration cases. Markers denote the four trace types (top and bottom traces of the left and right bushings); the dashed line is the 1:1 reference. The overall coefficient of determination is $R^{2}=0.787$.

Figure 16 summarizes the overall predictive accuracy of the extended pressure–strain wear model. The parity plot compares the predicted and measured wear depths for all 220 axial sample points from the five 30-min calibration cases. The dashed line represents the ideal 1:1 agreement, and the four marker types correspond to the four trace positions. Most data points are distributed close to the 1:1 line, indicating that the model reproduces both the magnitude and the spatial distribution of the measured wear. The majority of points are clustered in the low-wear region below 6 μm, corresponding mainly to the central and lightly loaded portions of the traces. The high-wear points above 10 μm are associated with edge-contact peaks, especially on the right bushing, and these points are also predicted without a strong systematic bias toward overprediction or underprediction.

The trace-wise coefficients of determination range from 0.71 for the right-bushing top trace to 0.85 for the right-bushing bottom trace. This indicates that the model accuracy is not controlled by a single favorable trace but remains reasonably consistent across the four measurement positions. The remaining scatter is concentrated in a limited number of points, mainly from cases in which the measured wear deviates from the pressure-supported edge-wear pattern. These deviations likely reflect specimen-to-specimen variability, local running-in effects, and contact-state changes that are not fully captured by the static FE pressure field. Overall, Figure 16 confirms that the extended pressure–strain model provides a balanced fit over the full range of measured wear depths and offers a quantitatively improved description of bushing wear under the nominal 34.3° loading condition.

To assess the stability of the calibration and the relative importance of the individual coefficients, the behaviour of the fitted coefficients was examined. This indicates that the dominant pressure term governs the overall fit, whereas the strain-coupling parameters and the constant offset are comparatively weakly constrained, so that perturbing them changes the global error only slightly while several converge to similar values. This shows that the plain pressure dependence carries most of the predictive information, which supports the parsimonious Archard form, while the additional strain terms mainly refine the prediction of the edge peaks.

Several simplifying assumptions in the finite element model bound the accuracy of the predicted contact pressures and, in turn, of the wear predictions. First, the friction coefficient was held constant at 0.5, whereas in practice it varies with local sliding speed, temperature, and surface state; a higher or spatially varying coefficient would alter the tangential traction and could shift and sharpen the edge-pressure peaks. Second, all materials were treated as linear elastic, so the localized plastic flow expected at the most heavily loaded edges is not represented. This tends to overstate the peak pressure and understate the contact-patch width, which partly explains the residual underestimation of the sharpest measured peaks. Third, the crankshaft was treated as effectively rigid relative to the bushing, so shaft bending and the associated migration and broadening of the contact patch under load are not captured. This is the principal reason the static pressure field cannot follow the late-stage, deepening-dominated wear at 60 and 90 min. These assumptions were adopted to keep the model tractable and to isolate the pressure-driven wear response. A velocity- and temperature-dependent friction law, an elastic–plastic material model, and a flexible-shaft formulation with periodic updating of the worn geometry are identified as the principal directions for improving predictive accuracy.

## 5. Conclusions

The main findings of this study are summarized as follows.

1. Bushing wear under high-speed press conditions was governed by a deterministic, geometry-driven edge-contact mechanism rather than by uniform sliding. Material removal formed a diagonally paired pattern at specific axial edges, and this pattern persisted across the 30, 60, and 90 min tests. The wear also evolved in two stages. The worn bands first broadened laterally between 30 and 60 min and then deepened sharply between 60 and 90 min at the same axial locations. Once the contact site was established, wear advanced mainly by deepening rather than by moving to new locations. Because wear concentrates at a few predictable edge zones, the service life of press bushings can be estimated from these critical sites alone. This lets maintenance intervals and clearance limits be set from a small number of locations rather than from the whole bore.
2. The finite element contact analysis confirmed the mechanical origin of this behavior. Contact pressure concentrated in narrow edge bands, while the central bore carried almost no load. The highest pressure, 72.6 MPa at the front edge of the right bushing, coincided with the measured maximum-wear location. Using this pressure field, the plain spatial Archard model (Equation (3)) reproduced the general pressure–wear trend. It converged to $K^{*}=0.609$ and α=0.929, with an RMSE of 1.42 μm and an MAE of 0.81 μm. The exponent near unity indicates a near-classical Archard response, although the model underestimated the sharpest edge peaks.
3. Adding the multiplicative stretch-ratio coupling (Equation (4)) improved the fit. It reduced the RMSE to 0.98 μm and the MAE to 0.57 μm and raised $R^{2}$ to 0.787. The improvement came mainly from better prediction of the high-pressure edge peaks.

The present study has three main limitations. First, the finite element validation applies only to the 30 min condition. The 60- and 90 min results are experimental observations and were not independently reproduced by the model. Second, wear was driven by a single static contact-pressure field obtained under a rigid-shaft assumption. This field cannot follow the evolving worn geometry or the shaft deflection that occur during operation. Third, the extended pressure–strain model contains several weakly constrained coefficients. These added parameters should be treated as empirical refinement rather than as independently identified physical constants.

These limitations define the main directions for future work. To address the first, the finite element calibration will be extended to the 60- and 90 min durations, so that the model is validated over the full test range. To address the second, a flexible-shaft model with periodic updating of the worn geometry will be introduced. This will capture shaft deflection, contact-patch migration, and the late-stage deepening seen at the longer durations. To address the third, a larger experimental dataset and complementary deformation measurements will be used to constrain the coefficients more firmly and to give them a clearer physical meaning.

In summary, the main contribution of this work is a physics-informed framework that links FEA-derived contact pressure to measured wear depth for copper-alloy bushings under high-speed press conditions. By calibrating a spatial Archard model against profilometry data, the framework predicts both where wear forms and how fast it grows from the contact-pressure field alone. The approach is not limited to the bushing. Every loaded interface in a press machine can be described by its own contact-pressure distribution. The calibrated pressure-to-wear relationship and the accelerated-test procedure can therefore be transferred to other components, such as crankshaft journals, slide gibs, and connecting-rod bearings. The present results thus form a building block toward whole-machine life prediction for high-speed press systems.

## Figures and Tables

**Table 1:** Chemical composition and mechanical properties of the copper alloy bushing material.

Category	Parameter	Specification	Measured Value
Chemical Composition (wt%)	Cu	85.0–95.0	88.01
	Sn	7.0–9.0	8.52
	Zn	1.0–5.0	2.58
	P	≤0.1	0.063
Mechanical Properties	Tensile Strength (MPa)	≥150	375
	Hardness (HB)	≥60	81

**Table 2:** Main specifications of the experimental components.

Component	Model/Material	Dimensions/Capacity	Additional Information
Motor	HG-JR503	5 kW	AC servo motor
Crankshaft	S45C steel	Diameter: 29.9 mm; Length: 277 mm	Machined solid shaft
Bushing	Special bronze casting alloy	Outer ⌀: 40 mm; Inner ⌀: 30 mm; Length: 50 mm	Plain bearing type
Load cell	CSMN-1T	4 units × 100 kgf	Compression type; Curiosity Technology, Paju, Republic of Korea
Pulley	—	63.5 mm/254 mm	Transmission ratio: 4:1
Servo driver	MR-J4	—	Mitsubishi Electric Corporation, Tokyo, Japan; configured via MR Configurator2 (Ver. 1.21X)
Load-cell amplifier	HX711	—	Load-cell signal amplification
Data-acquisition board	Arduino Uno	—	Arduino, Italy; load recorded at 10 Hz
Profilometer	SJ-410	Resolution: 0.001 μm	Mitutoyo Corporation, Kawasaki, Japan; axial wear profile
V-belt	A type	—	Power transmission

**Table 3:** Calibrated coefficients and fitting accuracy of Equations (3) and (4) using the combined 30-min dataset under the AVG load-cell calibration.

Model	Coefficients	*R*_2_	RMSE (μm)	MAE (μm)
Plain Archard, Equations (3)	$K^{*}=0.609$; α=0.929	0.558	1.42	0.81
Extended pressure–strain model, Equations (4)	$a_{0}=0.738\;\mu m$; $a_{1}=-1.116$; $a_{2}=1.210$; $k_{1}=2.095$; $k_{2}=2.075$; n=0.9997	0.787	0.98	0.57

## Data Availability

The original contributions presented in this study are included in the article. Further inquiries can be directed to the corresponding author.

## Abbreviations

The following abbreviations are used in this manuscript:

AVG	Time-averaged (average)
CPRESS	Contact Pressure
DAQ	Data Acquisition
EMI	Electromagnetic Interference
FEA	Finite Element Analysis
FEM	Finite Element Method
LE	Logarithmic strain (maximum-principal, true strain)
MAE	Mean Absolute Error
PLC	Programmable Logic Controller
RMSE	Root Mean Square Error
SNR	Signal-to-Noise Ratio
SPM	Strokes Per Minute
